# Emergence of Marburg virus: a global perspective on fatal outbreaks and clinical challenges

**DOI:** 10.3389/fmicb.2023.1239079

**Published:** 2023-09-13

**Authors:** Shriyansh Srivastava, Deepika Sharma, Sachin Kumar, Aditya Sharma, Rishikesh Rijal, Ankush Asija, Suraj Adhikari, Sarvesh Rustagi, Sanjit Sah, Zahraa Haleem Al-qaim, Prashant Bashyal, Aroop Mohanty, Joshuan J. Barboza, Alfonso J. Rodriguez-Morales, Ranjit Sah

**Affiliations:** ^1^Department of Pharmacology, Delhi Pharmaceutical Sciences and Research University (DPSRU), New Delhi, India; ^2^Department of Pharmacy, School of Medical and Allied Sciences, Galgotias University, Greater Noida, India; ^3^Division of Infectious Diseases, University of Louisville, Louisville, KY, United States; ^4^WVU United Hospital Center, Bridgeport, WV, United States; ^5^Manipal College of Medical Sciences, Pokhara, Nepal; ^6^School of Applied and Life Sciences, Uttaranchal University, Dehradun, Uttarakhand, India; ^7^Global Consortium for Public Health and Research, Datta Meghe Institute of Higher Education and Research, Jawaharlal Nehru Medical College, Wardha, India; ^8^Department of Anesthesia Techniques, SR Sanjeevani Hospital, Siraha, Nepal; ^9^Al-Mustaqbal University College, Hilla, Iraq; ^10^Lumbini Medical College and Teaching Hospital, Kathmandu University Parvas, Palpa, Nepal; ^11^Department of Clinical Microbiology, All India Institute of Medical Sciences, Gorakhpur, Uttar Pradesh, India; ^12^Escuela de Medicina, Universidad César Vallejo, Trujillo, Peru; ^13^Master Program on Clinical Epidemiology and Biostatistics, Universidad Científica del Sur, Lima, Peru; ^14^Gilbert and Rose-Marie Chagoury School of Medicine, Lebanese American University, Beirut, Lebanon; ^15^Department of Microbiology, Tribhuvan University Teaching Spital, Institute of Medicine, Kathmandu, Nepal; ^16^Department of Microbiology, Dr. D. Y. Patil Medical College, Hospital and Research Centre, Dr. D. Y. Patil Vidyapeeth, Pune, Maharashtra, India; ^17^Department of Public Health Dentistry, Dr. D. Y. Patil Dental College and Hospital, Dr. D. Y. Patil Vidyapeeth, Pune, Maharashtra, India

**Keywords:** filovirus, pathogenesis, Marburg virus, epidemic, vaccine, treatment

## Abstract

The Marburg virus (MV), identified in 1967, has caused deadly outbreaks worldwide, the mortality rate of Marburg virus disease (MVD) varies depending on the outbreak and virus strain, but the average case fatality rate is around 50%. However, case fatality rates have varied from 24 to 88% in past outbreaks depending on virus strain and case management. Designated a priority pathogen by the National Institute of Allergy and Infectious Diseases (NIAID), MV induces hemorrhagic fever, organ failure, and coagulation issues in both humans and non-human primates. This review presents an extensive exploration of MVD outbreak evolution, virus structure, and genome, as well as the sources and transmission routes of MV, including human-to-human spread and involvement of natural hosts such as the Egyptian fruit bat (*Rousettus aegyptiacus*) and other *Chiroptera species*. The disease progression involves early viral replication impacting immune cells like monocytes, macrophages, and dendritic cells, followed by damage to the spleen, liver, and secondary lymphoid organs. Subsequent spread occurs to hepatocytes, endothelial cells, fibroblasts, and epithelial cells. MV can evade host immune response by inhibiting interferon type I (IFN-1) synthesis. This comprehensive investigation aims to enhance understanding of pathophysiology, cellular tropism, and injury sites in the host, aiding insights into MVD causes. Clinical data and treatments are discussed, albeit current methods to halt MVD outbreaks remain elusive. By elucidating MV infection’s history and mechanisms, this review seeks to advance MV disease treatment, drug development, and vaccine creation. The World Health Organization (WHO) considers MV a high-concern filovirus causing severe and fatal hemorrhagic fever, with a death rate ranging from 24 to 88%. The virus often spreads through contact with infected individuals, originating from animals. Visitors to bat habitats like caves or mines face higher risk. We tailored this search strategy for four databases: Scopus, Web of Science, Google Scholar, and PubMed. we primarily utilized search terms such as “Marburg virus,” “Epidemiology,” “Vaccine,” “Outbreak,” and “Transmission.” To enhance comprehension of the virus and associated disease, this summary offers a comprehensive overview of MV outbreaks, pathophysiology, and management strategies. Continued research and learning hold promise for preventing and controlling future MVD outbreaks.

**GRAPHICAL ABSTRACT:**
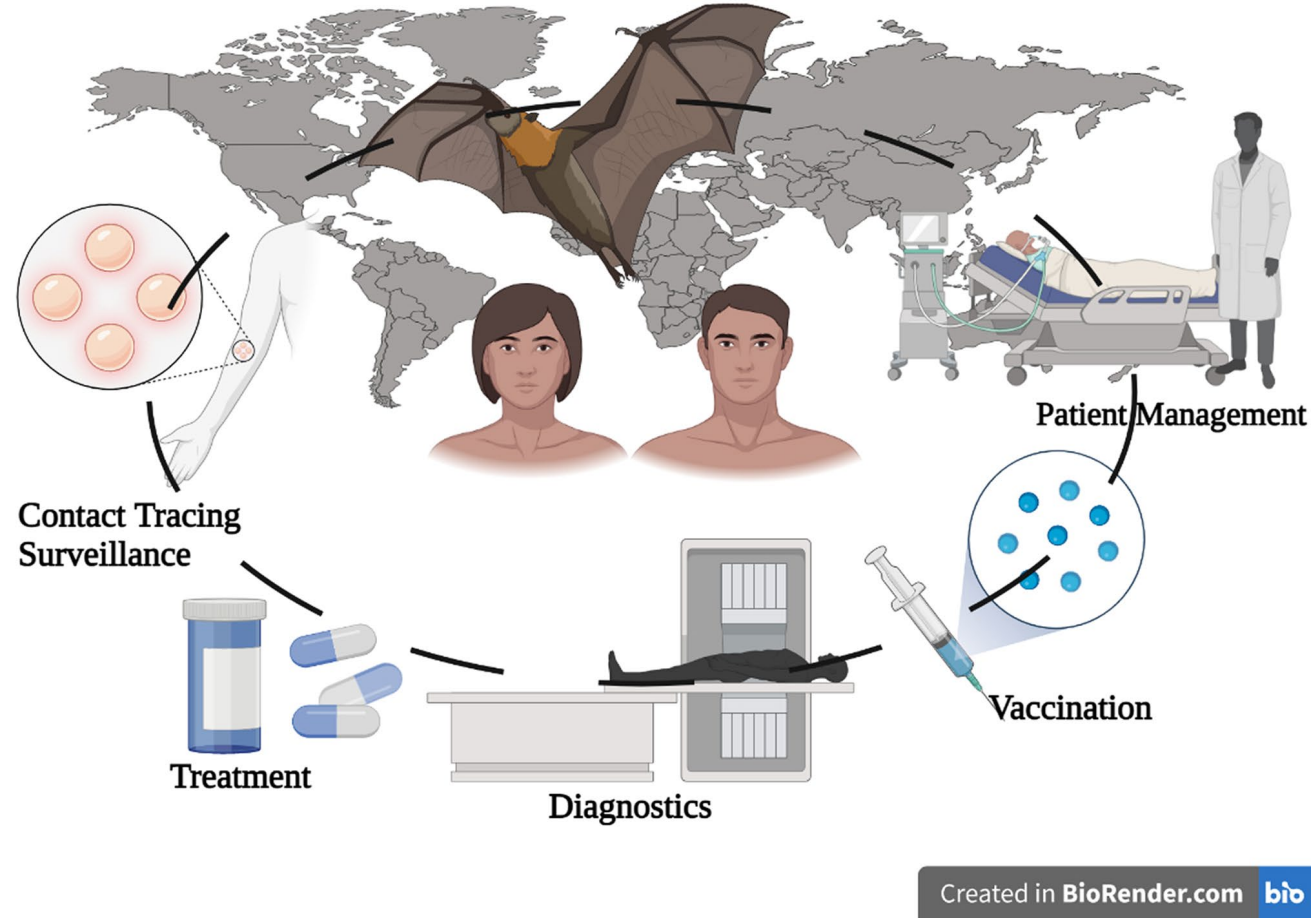

## 1. Introduction

The Marburg virus (MV) has emerged as a significant global threat, instigating multiple fatal outbreaks with a considerable mortality rate since its discovery and characterization in 1967. Its initial appearance spanned regions including Marburg, Frankfurt (Germany), Yugoslavia (now Serbia), and Belgrade, with Africa primarily bearing the brunt of its impact (Brauburger et al., 2012). According to the National Institute of Allergy and Infectious Diseases, the main cause of MVD is Marburg virus (Bente et al., 2009). The virus’s nomenclature stems from the city where it is devastating effects were most pronounced, resulting in seven deaths among 31 early patients (Brauburger et al., 2012). Investigation subsequently unveiled the virus’s origin in imported Ugandan green African monkeys (Gear et al., 1975). Contrary to the belief that MV posed a lesser threat, significant outbreaks in the Democratic Republic of the Congo (DRC) from 1998 to 2000 and in Angola from 2004 to 2005 demonstrated a fatality rate of 83% and 90%, respectively, dispelling this notion (Bausch et al., 2006; Qiu et al., 2014). Different variants were connected to the outbreak in the DRC, but just one version spread from person to person in Angola (Bertherat et al., 1999; Bausch et al., 2006). By 2008, reported MV cases surged to 452, with 368 confirmed deaths, raising concerns of underreporting (Brauburger et al., 2012). Despite receiving less media attention than its *Filoviridae* counterparts, MV’s elevated fatality rates underscore its significance. Recent cases in Guinea, Ghana, and the ongoing Equatorial Guinea outbreak accentuate the necessity for continuous surveillance and containment. The latter reported a swift succession of deaths, and Tanzania also reported fatalities attributed to the virus, highlighting the ongoing threat (Manohar et al., 2023). MVD’s impact spans hemorrhagic fever and organ dysfunction in both humans and non-human primates, affecting the liver, spleen, brain, kidneys, and causing coagulation anomalies (Mehedi et al., 2011; van Paassen et al., 2012). As a member of the Marburg virus genus within the *Filoviridae* family and *Mononegavirales* order, MV comprises a distinct species (Bukreyev et al., 2014). Its zoonotic connection to the Egyptian fruit bat (*Rousettus aegyptiacus*) and human-to-human transmission parallel other Ebola viruses, such as Sudan virus, *Bundibugyo virus*, and EBOV. Ongoing research has elucidated MV’s natural sources, including *Hipposideros caffer*, in tandem with *Rousettus aegyptiacus* (Towner et al., 2009). With an average incubation period of 5 to 10 days, ranging from 3 to 21 days, the virus infiltrates immune cells like monocytes, macrophages, and dendritic cells via compromised skin or mucosal surfaces. It initiates replication in the spleen, liver, and secondary lymphoid organs before propagating to hepatocytes, endothelial cells, fibroblasts, and epithelial cells. Additionally, it thwarts interferon type I (IFN-1) production (Yu et al., 2021). This review explores the evolutionary course of MVD outbreaks, succinctly delineating the virus’s structure and genome, elucidating MV’s origins, and detailing its diverse transmission modes among humans and non-human sources. We comprehensively examine MVD’s genesis through scrutinizing pathophysiology, cellular tropism, immune evasion, and critical host injury sites. Additionally, this overview encompasses current clinical data and treatments, underscoring the vital need for robust research to foster effective drugs and vaccines. By deepening comprehension of the virus’s historical progression and infection mechanisms, this review strengthens defenses against MV disease, driving the innovation of therapeutic agents and vaccines, thus supporting future researchers in countering this enduring health threat.

## 2. Mutational analysis and genome composition

**Figure 1:**
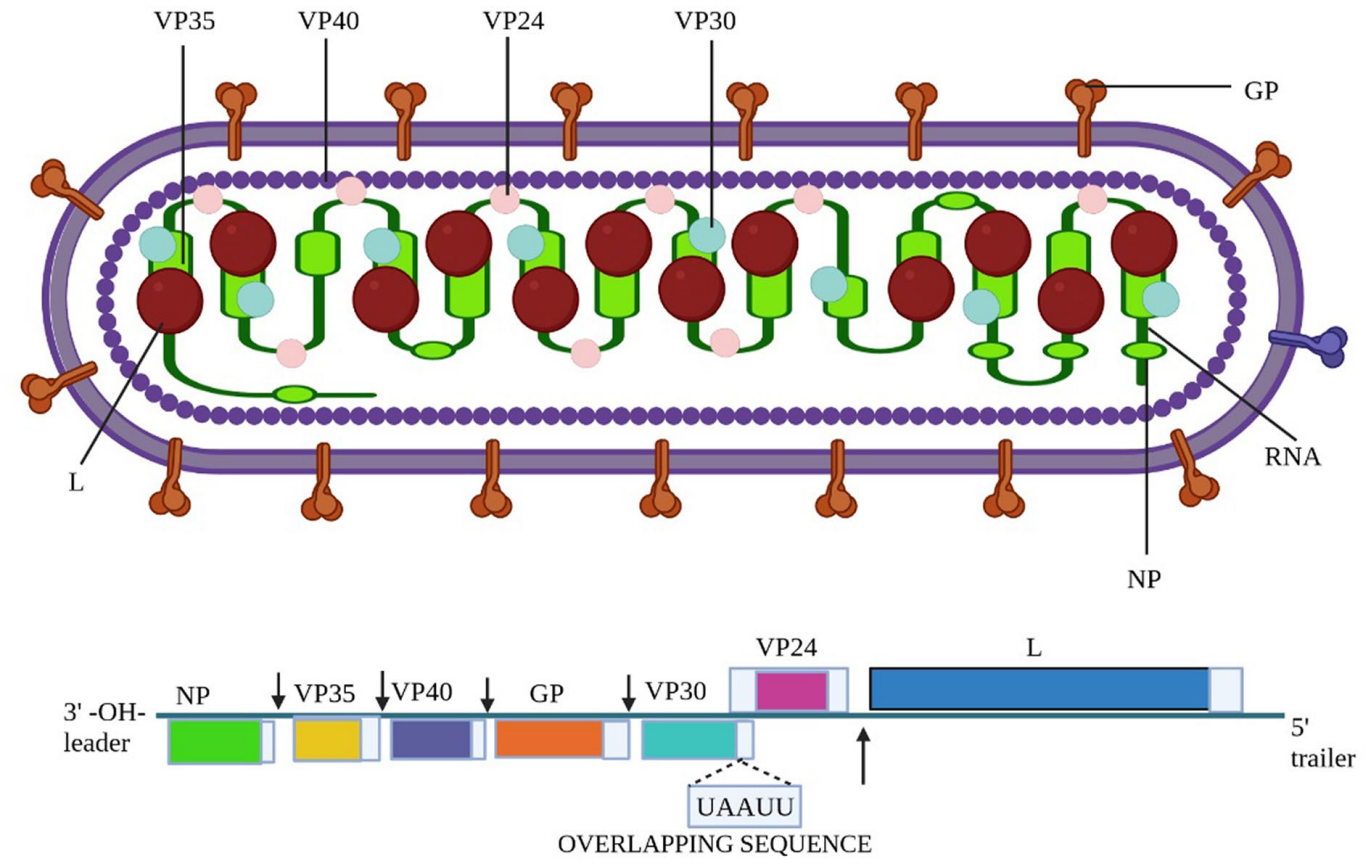
The structure of the Marburg virus and the organization of its genome are depicted in the figure. The upper portion of the figure shows the structure of the virus and identifies the structural proteins. The genomic organization of the seven-gene Marburg virus strain is depicted in the lower section of the figure, which has been crudely scaled. Light blue boxes indicate non-coding regions, whereas colored boxes depict the coding sections of genes. Except for the overlap between VP24 and VP30, which is depicted as a black triangle, the genes are separated by intergenic regions, as shown by the black arrows. At the ends, the 3′ and 5′ trailer sequences are also displayed. Bio render software was used to create this figure (Abir et al., 2022).

The Marburg virus (MV) exhibits an enveloped and pleomorphic structure, displaying uniform diameter but variable length filamentous, non-segmented, rod-like, cobra-like, circular/annular, and branched particles (Chakraborty et al., 2022; Islam et al., 2023a). Its viral genome encompasses seven open reading frames (ORFs), namely nucleoprotein (NP), virion protein 35 (VP35), VP40, VP30, VP24, glycoprotein (GP), and large viral polymerase, all characterized as single-stranded negative-sense RNA (-ssRNA; Zhao et al., 2022). The non-coding regions of these seven genes contain cis-acting elements implicated in DNA replication, transcription, and packaging (Feldmann et al., 1992; Sanchez et al., 1993). The 3′ and 5′ ends of these genes possess unusually long non-coding nucleotide sequences and highly conserved transcription start and stop signals (Feldmann et al., 1992; Sanchez et al., 1993). Intergenic regions separate all MV genes except two, ranging from 4 to 97 nucleotides in length, and the transcription start and stop signals of the VP24 and VP30 genes share an overlapping sequence of five nucleotides (UAAUU). The nucleocapsid complex, comprising structural proteins NP, VP35, VP30, and L, envelops the MV genome (Becker et al., 1998). VP35, acting as a polymerase cofactor, and L, functioning as an RNA-dependent RNA polymerase, are essential for replication and transcription of viral genomes (Mühlberger et al., 1999). The host-derived membrane layer of MV is regularly spiked, where highly glycosylated protein (GP) plays a key role in binding to receptive host cells (Feldmann et al., 1991). VP40, responsible for budding and binding to the matrix and nucleocapsid, constitutes the inner matrix of a virion (Kolesnikova et al., 2004; Swenson et al., 2004). The interaction of the protein VP24 with the membrane NP and other cell membranes is vital for the release of virion progeny. Table 1 provides an overview of the characteristics and functions of the proteins in MV (Bamberg et al., 2005). Phylogenetic analysis of Marburg virus sequences has been conducted to ascertain sequence cohesion and identify splits between sequences of particular interest and well-known sequences that exhibit unexpectedly deep divergence (Peterson and Holder, 2012). From cDNA clones made from genomic RNA and mRNA, the first 3,000 nucleotides of the Marburg virus genome were identified (Sanchez et al., 1992). There is up to 21% nucleotide diversity among previously characterized East African strains of the Marburg virus, according to partial Marburg virus RNA sequence study (Towner et al., 2006). Serial passages of Marburg virus resulted in a single mutation in the region encoding the glycoprotein (GP; Alfson et al., 2018). To clarify the relationship between various Marburg virus strains, phylogenetic analysis of full-length or partial genomes of Marburg viruses obtained from people or bats has been carried out (Towner et al., 2009). Phylogenetic analysis showed the 2021 Guinean Marburg virus’ relationship to strains from the 2004–2005 Marburg virus outbreak in Angola, which are related to Marburg virus sequences obtained from bats in Sierra Leone (2017–2018). The viral genome sequence of the 2021 Guinean Marburg virus was recovered to 99.3% (Magassouba, 2021). Sequential mouse passaging and cell-culture adaption during deep sequencing of the Marburg virus genome have shown significant changes over time (Wei et al., 2017). An analysis was performed on the sequencing data to identify sites in viral mRNAs (Shabman et al., 2014). Structural and functional studies on the Marburg virus GP2 fusion loop have also been conducted (Liu et al., 2015). Marburg virus has been subjected to mutational and phylogenetic analysis, and the genome has been sequenced to identify changes over time shown in Figure 1 (Mühlberger, 2007).

**Table 1:** Marburg virus: genes, proteins, characteristics, and functions.

S. no	Gene (sequence)	Protein (abbreviation)	Description	Amino acid	Functions	Gene length	References
1.	NP	Nucleoprotein (NP)	Binds to VP35 protein, VP40, VP30, and VP24, and is a component of the RNP complex. It also undergoes phosphorylation and homo-oligomerizes to form a helical polymer. Additionally, it is the second-most prevalent protein found in virions.	695	Creation of NC and cellular inclusion body; Encapsidation of RNA genome as well as antigenome; Replication and transcription; Budding	2,796/2,088	DiCarlo et al. (2011), Brauburger et al. (2015), and Liu et al. (2017)
2.	VP35	Viral protein 35	RNP complex components that bind to dsRNA, NP, and L and homo-oligomerize. They are also weakly phosphorylated.	329	Formation of NC; RdRp cofactor; Replicase transcriptase cofactor; IFN antagonist	1,557/990	Bale et al. (2012), Brauburger et al. (2015), Edwards et al. (2016), and Bruhn et al. (2017)
3.	VP40	Viral Protein 40	It homo-oligomerizes to form dimers, circular hexamers, and octamers, binds ssRNA and VP35, and is one of the most common proteins in virions and infected cells.	303	Matrix component: Negative regulator of transcription and replication; Budding and host adaption; regulation of the morphogenesis of the virion and egress; hinders JAK–STAT pathway	1,405/912	Kolesnikova et al. (2012), Brauburger et al. (2015), Koehler et al. (2018), and Amiar et al. (2021)
4.	GP	Glycoprotein (GP1,2)	Uses GP_1_ and GP_2_ subunits to create heterodimers; the mature protein is found as a trimer of GP_1,2_ heterodimers; can insert into membranes; Acylated, substantially N- and O-glycosylated and phosphorylated. Class I fusion and type I transmembrane protein, along with ADAM17, convert GP_1,2_ into soluble GP_1,2._	681	Attachment of virions to susceptible cells using cellular attachment factor: determination of cell and tissue tropism; Receptor binding; induction of virus-cell membrane; Tetherin antagonist;	2,846/2,046	Carette et al. (2011) and Brauburger et al. (2015)
5.	VP30	Viral protein 30 activator	RNP complex components with high phosphorylation, ssRNA, NP, and L binding, as well as a zinc binding domain.	281	Formation of NC; Initiation, reinitiation and antitermination and enhancement of transcription	1,249/846	Enterlein et al. (2006), Wenigenrath et al. (2010), and Wan et al. (2017)
6.	VP24	Viral protein 24	Components of the RNP complex; homo-tetramerizes; connected with the hydrophobic membrane.	253	Formation and maturation of NC; Negative regulation of transcription; regulation of replication; virion morphogenesis regulatory function	1,287/762	Edwards et al. (2014), Page et al. (2014), Zhang et al. (2014), Brauburger et al. (2015), and Wan et al. (2017)
7.	L	Large protein (L)	RNP complex components that bind to VP35, VP30, genomic, and antigenomic RNA, as well as mRNA capping enzymes, and homodimerize.	2,331	Catalytic domain of RdRp; Replication of genome; Transcription of mRNA	7,745/69,96	Koehler et al. (2016) and Kirchdoerfer et al. (2017)

## 3. Epidemiology

### 3.1. MVD outbreak in 1967

In the European outbreak, most patients between 2 and 7 days after the onset of symptoms had non-itchy rash. The diseases were linked to three laboratories in different cities that had each received an infected cargo of African green monkeys. The first MVD outbreak, which was documented in 1967 and was brought on by laboratory workers in Germany and Serbia handling green African monkeys called grivets (*Chlorocebusaethiops*), was brought on by these imports. According to grivets’ reports, staff employees were primarily exposed to these sick wild animals’ meat and organs, which caused them to contract MV (Mehedi et al., 2011; Languon and Quaye, 2019). Seven patients died as a result of the sickness, which affected a total of 31 patients and was caused by 25 primary and 6 secondary infections (Deb et al., 2023).

### 3.2. MVD outbreak in 1975

In 1975, the MVD outbreak marked the second recorded instance of the disease and the initial occurrence in Africa. It took place in Johannesburg, South Africa, involving three cases and resulting in one fatality. The outbreak originated from a 20-year-old Australian man who had journeyed to Zimbabwe (then Rhodesia) and explored several bat-inhabited caves. After his return to Johannesburg, he exhibited symptoms such as fever, headache, myalgia, and vomiting, ultimately succumbing on 5 February 1975. His travel companion and a nurse who cared for him were secondary cases, both of whom survived through supportive treatment. Swift case isolation and contact tracing successfully contained the outbreak. While the source of infection remained unconfirmed, suspicion centered on the index case acquiring the virus through exposure to bats or their droppings within the caves (Gear et al., 1975; Abir et al., 2022).

### 3.3. MVD outbreak in 1980

In 1980, Kenya experienced the third documented outbreak of Marburg-virus disease. The initial patient contracted the infection in western Kenya, leading to the subsequent infection of a doctor in Nairobi who had close contact with the patient, ultimately resulting in severe haematemesis. However, no additional instances of transmission within medical settings were observed. Surveillance efforts in western Kenya did not uncover evidence of Marburg-virus disease, but they did indicate the potential existence of Ebola hemorrhagic fever (Smith et al., 1982).

### 3.4. MVD outbreak in 1987

In 1987, a single case of MVD outbreak emerged in Kenya, centering around a 15-year-old Danish boy who fell victim to the infection and subsequently passed away. The boy had encountered the virus within a cave inhabited by Egyptian fruit bats. This marked the inaugural documented instance of Ravn virus transmission, a near kin of the Marburg virus responsible for MVD. Effective containment measures were enacted through patient isolation and contact tracing, successfully preventing the emergence of secondary cases (Johnson et al., 1996).

### 3.5. MVD outbreak in 1998 and 2000

In both 1998 and 2000, there was a single MVD outbreak situated in Durba, Democratic Republic of the Congo (DRC). The affected individuals were gold miners who toiled within a mine known to be home to Egyptian fruit bats, the virus’s natural reservoir. This outbreak encompassed a total of 154 cases and tragically led to 128 fatalities, resulting in an 83% case fatality rate. Remarkably, this marked the initial occurrence of a substantial MVD outbreak and the primary instance of a combined outbreak involving Marburg virus and Ravn virus. These two closely related viruses both contribute to causing MVD (Colebunders et al., 2007; Towner et al., 2009).

### 3.6. MVD outbreak in 2004 and 2005

A second significant MV outbreak in the Uige region of Angola began in October 2004 and lasted until July 2005. The root cause of the epidemic, which later spread to other provinces, was discovered to be the death of a hospital employee in Uige. This epidemic has the highest number of illnesses and deaths linked to a single outbreak to date, with 252 cases of infection and 227 fatalities (a 90% mortality rate). An epidemic that occurred in Uganda in 2007 only resulted in four confirmed cases. The patients were Ibanda district workers at the Kitaka mine. The two workers contracted the disease after sharing a tent camp with the index case in the Kashoya-Kitomi Central Forest Reserve close to the mine. The fourth patient was working at the mine when the epidemic started, without any personal protective equipment (PPE). The mining adit was surrounded by bats, and the only personal protective equipment (PPE) accessible was a pair of gloves; masks, respirators, or goggles were not. The main source of infection was direct contact with bats or bat excretions. During this epidemic, MV was isolated from *Rousettus aegyptiacus*, and the first definite filovirus reservoir was discovered by sampling bats (Languon and Quaye, 2019).

### 3.7. MVD outbreak in 2012 and 2017

On 29 November 2012, the Ugandan Ministry of Health declared MV infection in Uganda. The Ugandan districts of Kabale, Ibanda, Mbarara, and Kampala have recorded about 15 fatalities and 8 probable cases (Deb et al., 2023). This outbreak in the Ibanda district occurred concurrently with the 2007 MV disease outbreak in the Kitaka mining region. As a result, *Rousettus aegyptiacus* bats were once more connected to the outbreak in 2012. It is interesting to note that the epidemic hit the *Rousettus aegyptiacus* bat population during the second half of the yearly viral cycle (Languon and Quaye, 2019). Additionally, the genome sequences of this MV strain and the MV strain that had previously been identified in Egyptian fruit bats were similar. A health worker contracted the disease and succumbed to it in Kampala, Uganda in 2014, where an epidemic also occurred (Nyakarahuka et al., 2017). In 2017, a new MV outbreak occurred in the Kween region of Uganda. The four family members who became MV-infected during this outbreak only had one survivor (Nyakarahuka et al., 2019). Nevertheless, extensive studies are still being conducted because the clinical evidence for this outbreak is still insufficient.

### 3.8. MVD outbreak in 2021

Last but not least, Guinea saw its most recent outbreak in August 2021, which was eventually contained in September 2021. One man became ill and died during this time, but the strain is still unclear (Aborode et al., 2022; Makenov et al., 2023; WHO, 2023a).

### 3.9. MVD outbreak in 2023

**Figure 2:**
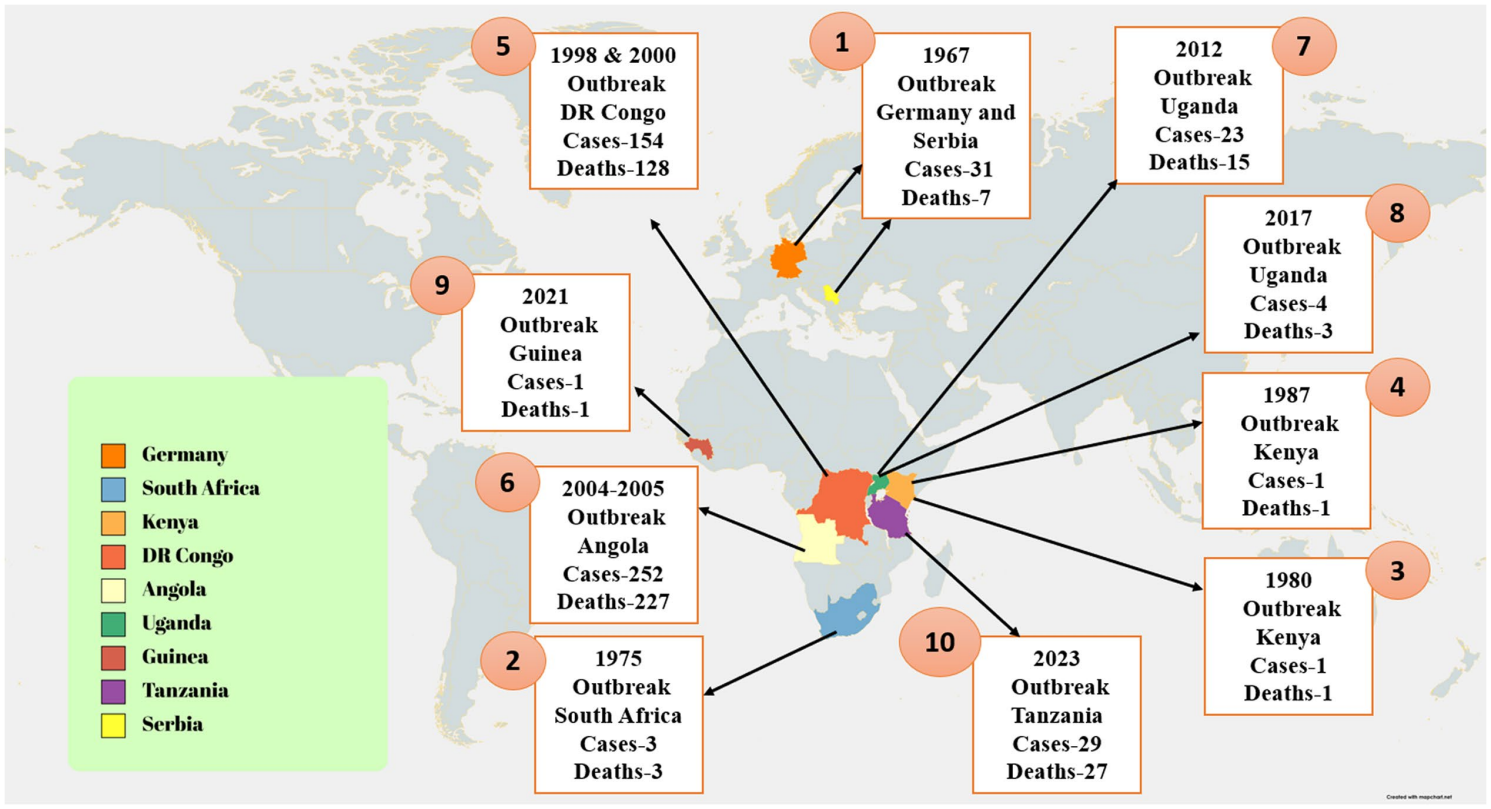
The image shows the distribution of confirmed Marburg virus disease (MVD) cases and associated fatalities. The image illustrates that the MVD outbreak in Tanzania was confined to a distinct region, with reported cases concentrated in the Kigoma region. Additionally, the image underscores the ongoing MVD outbreak in Equatorial Guinea, characterized by a broader geographical spread of cases. Moreover, the image also denotes further instances of MVD outbreaks across different African regions (Abir et al., 2022).

Between 7 January and 7 February 2023, two villages in the Nsock Nsomo district of the Ro Muni area in the eastern Kie-Ntem province experienced at least eight fatalities. The affected individuals presented symptoms such as fever, weakness, vomiting, and bloody diarrhea. In two cases, skin lesions and otorrhagia were also observed. On 9 February 2023, health authorities collected blood samples from eight contacts and submitted them to the Centre Interdisciplinary de Recherches Médicales de Franceville (CIRMF) in Gabon. However, real-time polymerase chain reaction (RT-PCR) testing at CIRMF yielded negative results for the presence of Marburg and Ebola viruses (WHO, 2023a). On 12 February 2023, additional blood samples were obtained from different contacts and sent to the Institute Pasteur in Dakar, Senegal. RT-PCR testing confirmed one of these samples to be positive for the Marburg virus. The patient associated with this confirmed case displayed symptoms of fever, bloodless vomiting, bloody diarrhea, and convulsions, ultimately succumbing to the infection on 10 February 2023, at Ebebiyin District Hospital. This case was linked to four other deceased cases originating from one of the villages in the Nsoc-Nsomo District. As of 21 February 2023, a total of nine cases have been reported, consisting of one confirmed, four probable, and four suspected cases, all of which resulted in fatalities. Health workers have not been affected, and 34 contacts remain under surveillance. After the initial outbreak, on 13 March 2023, two individuals from Kié-Ntem province, and on 15 March 2023, one person from Litoral province, tested positive for the Marburg virus through RT-PCR. On 18 and 20 March, three more laboratory-confirmed cases were reported from Litoral province, and on 20 March, two additional cases were identified in Centre Sur province. The extensive geographical spread of the infections and the uncertain epidemiological situation in Centre Sur Province raise concerns that the virus may be spreading undetected within the community (WHO, 2023a). Table 2 represents different outbreak of Marburg (Figure 2).

**Table 2:** Outbreaks of Marburg virus (MV).

MVD outbreak	Location	Transmission	Number of deaths	Number of cases	Case fatality rate	Mortality rate	References
1967	Germany, Serbia	Contact with grivets	7	31	22%	22.6%	Mehedi et al. (2011), Languon and Quaye (2019), and Deb et al. (2023)
1975	South Africa	Person-to-person	3	3	100%	100.0%	Gear et al. (1975) and Abir et al. (2022)
1980	Kenya	Person-to-person	1	1	100%	100.0%	Smith et al. (1982)
1987	Kenya	Unknown	1	1	100%	100.0%	Johnson et al. (1996)
1998 and 2000	Democratic Republic of the Congo	Unknown	128	154	83%	83.1%	Towner et al. (2009) and Colebunders et al. (2007)
2004–2005	Angola	Person-to-person	227	252	90%	90.1%	Languon and Quaye (2019)
2012	Uganda	Contact with *Rousettus aegyptiacus* bats	15	23	60%	65.2%	Deb et al. (2023)
2017	Uganda	Unknown	3	4	75%	75.0%	Languon and Quaye (2019) and Nyakarahuka et al. (2019)
2021	Guinea	Unknown	1	1	100%	100.0%	Nyakarahuka et al. (2019) and WHO (2023a)
2023	Equatorial Guinea, Tanzania,	Unknown	27	29	93%	86.0%	WHO (2023a)

### 3.10. Socioeconomic impact of MVD outbreaks

The socioeconomic impact of MVD outbreaks can significantly affect countries with weaker economies, leading to inadequate containment and management (Manohar et al., 2023). Epidemics can interact at the host population, influencing one another’s severity and trajectories when they co-occur (Reuben and Abunike, 2023). MVD outbreaks can result in fatalities and socioeconomic consequences, including loss of tourism (Nyakarahuka et al., 2017). Comprehensive research of MVD is necessary given its link to MV infection and the disease’s high fatality rate of up to 90% (Abir et al., 2022). The Filoviridae family, including MV, has caused significant loss of human and animal lives (Languon and Quaye, 2021). Past outbreaks have exhibited varying case fatality rates, ranging from 24% to 88%, contingent upon virus strain and case management (WHO, 2021a). According to the World Health Organization (WHO), MVD is a highly contagious disease that can cause hemorrhagic fever and has a death rate of up to 88%. The WHO advises treatment of symptoms, oral or intravenous rehydration, and supportive care to increase survival. Although continuing analyses of prospective remedies, such as blood products, immunological therapies, and pharmacological therapies, are being conducted, there is currently no known cure for MVD (WHO, 2023b). Case management, surveillance, contact tracing, a well-functioning laboratory service, safe and respectable burials, and social mobilization are only a few of the actions that are needed to suppress outbreaks. Community involvement is also essential for effective epidemic control (WHO, 2021a).

## 4. Source and transmission of Marburg virus

**Figure 3:**
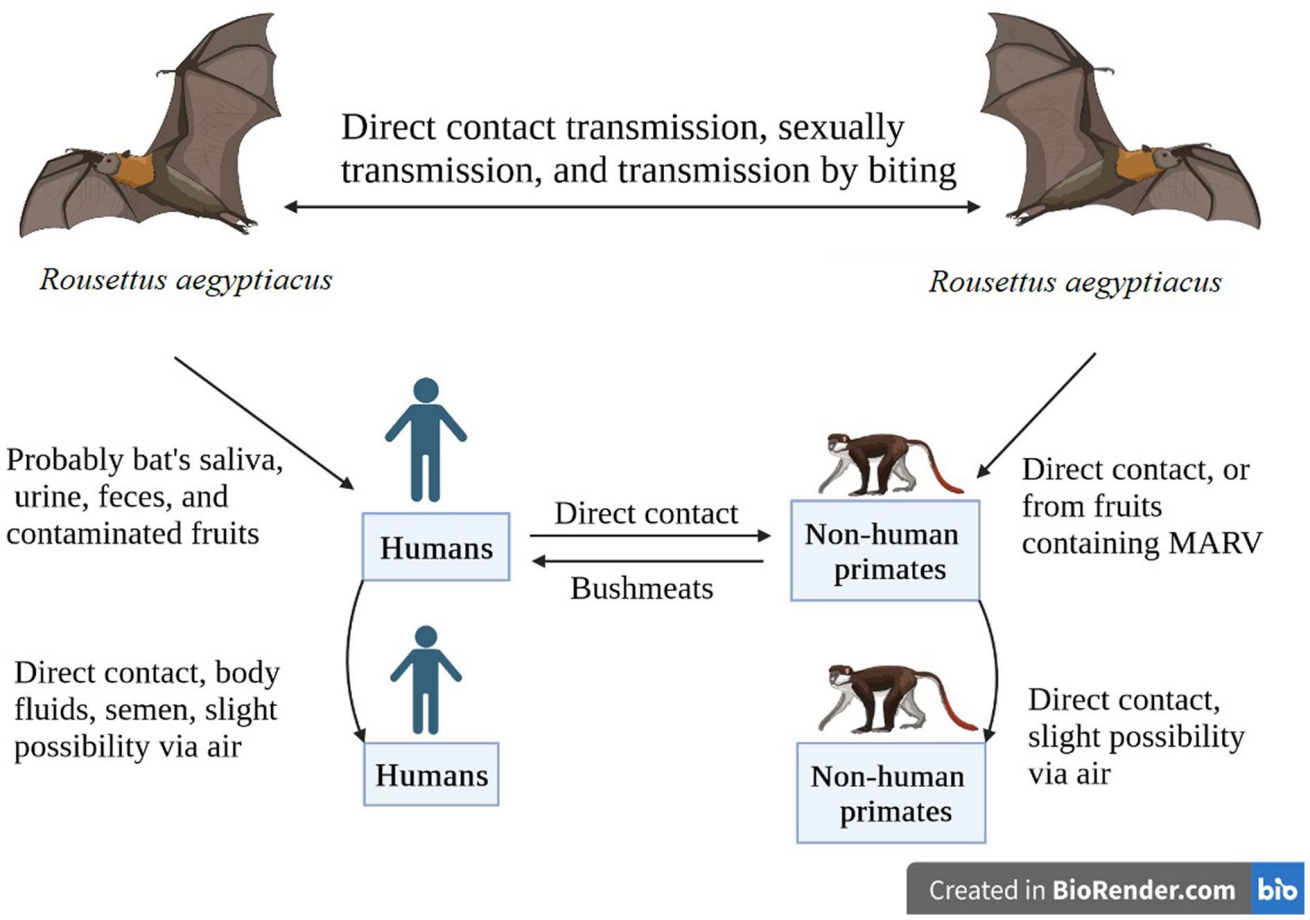
African fruit bats serve as reservoirs for the Marburg virus, which is conveyed by direct contact, sexual contact, or biting. Humans and non-human primates can contract the virus through viral-contaminated fruit consumption or direct contact with the reservoir hosts. Disease transmission can also occur through direct contact between NHPs and humans, or from NHPs to humans through bushmeat consumption. The image was created using Bio render software.

The fruit bat species *Rousettus aegyptiacus* is the most important natural source of MV infection. In addition, some *Chiroptera and Hipposideros caffer* may act as infectious agents. There are several ways in which MV strains can be transmitted from bat to bat. Recent research found MV in rectal, oral, and urine samples from infected bats as well as in blood and oral samples from bats that had come into contact with humans. According to this research, MV is horizontally transmitted from infected bats to bat contacts (Schuh et al., 2017). The findings of the previous study showed that MV was present in the tissues of the lungs, intestines, kidneys, bladder, salivary glands, and female reproductive tract of immunized bats, supporting the hypothesis that MV transmission might occur (Amman et al., 2012) both vertically and horizontally in reservoirs. It has also been suggested that bats could spread the illness to one another by bites (Amman et al., 2015), sexual contact (Amman et al., 2012), or hematophagous arthropods (Schuh et al., 2017). Direct contact (injured skin or mucous membranes) with the blood and other bodily fluids of infected people (urine, saliva, feces, vomit, breast milk, amniotic fluid, and semen) or indirect contact with contaminated surfaces and materials, such as contaminated clothing, bedding, and medical equipment, are the two main methods of interpersonal transmission after infection. Infection may happen if sick people are buried (Schwartz, 2019). Infected animals, particularly bushmeat (such as monkeys, chimpanzees, forest antelopes, and bats), whether alive or dead, can also spread the disease to humans (Figure 3; WHO, 2021a). Bushmeat consumption has been linked to Ebola virus (EBOV) outbreaks (Malik et al., 2023). Because numerous bushmeat species, including chimpanzees and forest antelope, are vulnerable to virus multiplication and consumption following infection (Heeney, 2015), they are regarded as intermediate hosts. There is growing concern about the persistence of filovirus in the testis as a potential route of transmission. Experimental trials have identified persistent MV infection of the immunoprivileged testicular tubules in male monkeys (Coffin et al., 2018). During the 1967 MV outbreak, the first possible case of sexual transmission was found. Two months after the recovery of a male patient, symptoms appeared in his wife, which were confirmed by the detection of MV antigen in his semen (Feldmann, 2018). Simultaneous testing of nine other convalescents for MV did not detect virus or viral antigen (Slenczka, 2017). In pregnant women, filovirus infection is usually more severe than in nonpregnant women, which may be due to decreased immune function or placental involvement (Brainard et al., 2016). Based on case reports, viral titers have been found in placental tissue, suggesting that hematogenous transplacental transmission is the most typical route of fetal infection (Bebell and Riley, 2015). Despite the high rates of pregnancy mortality, there is no proof that pregnant women are more prone to contracting filovirus than other people (Jamieson et al., 2014). Pregnant women who have MVD have an increased risk of stillbirths and spontaneous abortions (Schwartz, 2019). Little information is available on the effects of MVD infection in infants. There have been reports of several MVD clusters in newborns, some of whom had very mild symptoms (Borchert et al., 2002). MV can be present in the blood, organs, and tissues of sick or recovering individuals, suggesting that the virus can be transmitted through transfusion and transplantation. Filoviruses can survive for a very long time in liquid or dried materials (Piercy et al., 2010). They are inactivated by gamma irradiation, heating to 60°C for 60–75 min, or boiling for 5 min and are sensitive to fatty solvents, sodium hypochlorite, and other disinfectants (Kuhn, 2008; Feldmann et al., 2019).

## 5. Clinical signs and disease progression

Marburg virus disease (MVD) has an incubation period of two to 21 days. According to the World Health Organization (WHO), high fever, severe malaise, and headache are some of the sudden symptoms associated with Marburg virus infection. Patients frequently complain of muscle pain and pains at the same time. Patients may experience severe watery diarrhea, cramping in the stomach, nausea, and vomiting on the third day. Up to a week may pass during the phase of diarrhea. Patients frequently exhibit a characteristic appearance during this stage that is characterized by sunken eyes, indifferent facial expressions, and extreme sluggishness. Within 2 to 7 days of the onset of symptoms, a non-itchy rash commonly develops (Elsheikh et al., 2023). Hemorrhagic signs often appear 5 to 7 days after the initial episode, with fatal cases showing several sites of hemorrhage. Bleeding from the gums, nasal passages, and vagina usually accompany fresh blood in vomitus and feces. The problem of spontaneous bleeding at venipuncture sites is particularly difficult. High fevers characterize the illness’s severe stage. Involvement with the central nervous system might result in confusion, irritation, and violence. In the later stages of the illness, occasional observations of orchitis, which is defined by inflammation of one or both testicles, have been made (around day 15). Most frequently, death happens 8 to 9 days after the onset, generally after a serious bout of blood loss and a subsequent shock (Elsheikh et al., 2023). Direct contact with the blood, secretions, organs, or other bodily fluids of infected people, as well as with surfaces and objects contaminated with these fluids, can result in human-to-human transmission of the Marburg virus. This contact can happen through cuts, scrapes, or other breaks in the skin or mucous membranes. Exposure to fruit bat species can also cause transmission (Elsheikh et al., 2023). An alternative classification of MV clinical features includes a “Generalization Phase,” which is characterized by flu-like symptoms and rash, an “Early Organ Phase,” which involves manifestations in specific organs, like encephalitis or hemorrhages, and a “Late Organ/Convalescence Phase,” which is characterized by multiorgan failure, shock, coma, and either death or recovery. These phases, in that order, correspond to the first 4 days, the next 9 days, and the time after day 13 (Asad et al., 2020).

### 5.1. Phase 1 (generalization phase)

Flu-like symptoms such a high fever (>40°C), a strong headache, chills, myalgias, and malaise appear during the generalization phase. The fifth day from the start of the disease is when this phase may last, after which there is a rapid attenuation. There have been reports of exhaustion, general malaise, appetite loss, nausea, vomiting, abdominal pain, and excessively watery diarrhea (Feldmann et al., 2013). Pharyngitis, conjunctivitis, enanthem, dysphagia, and dysphagia are additional frequent problems. Before developing into a maculopapular rash, a rash may also show up on the face, feet, and limbs in the middle to late stages of the generalization phase. Other symptoms include lymphadenopathy, leukopenia, and thrombocytopenia (Leroy, 2009).

### 5.2. Phase 2 (early organ phase)

A prolonged high temperature and other nonspecific symptoms define the early organ phase, which lasts 5 to 13 days following the onset of symptoms. Patients may also experience conjunctival infections, edoema, tiredness, dyspnea, viral exanthems, and aberrant vascular permeability (Klenk et al., 1999). Neurologic symptoms have also been characterized by patients as causing disorientation, encephalitis, irritability, psychosis, and aggressiveness (Borchert and Van der Stuyft, 2008). About 75% of patients experience hemorrhagic symptoms, including hematemesis, ecchymosis, melena, petechiae, bloody diarrhea, visceral hemorrhagic effusions, uncontrolled leaking from venepuncture sites, and mucosal bleeding. Additionally, complaints of bleeding from the nose, gingiva, and vagina have been made. The kidney, liver, and pancreas are a few of the damaged organs at this point in the illness. Additionally, most infected people displayed elevated serum activity (Kuhn, 2008).

### 5.3. Phase 3 (late organ/recuperative phase)

There are two distinct outcomes in the late stages of MV infection: either the patient enters a protracted period of recovery or the sickness is fatal. Eight to 16 days after the onset of the initial symptoms, death frequently occurs. Typically, multiorgan failure and shock are the two main causes of demise (Martini et al., 1968). The late organ phase begins in nonfatal cases on day 13 and lasts until day 20 and beyond as the illness develops. Acute metabolic anomalies including convulsions and severe dehydration can cause anuria and multiple organ failure in addition to harming the patient’s general health. At this time, orchitis has occasionally been identified. The neurological problems are still there currently. Women who are pregnant who have spontaneous.

## 6. Pathogenesis of MVD

**Figure 4:**
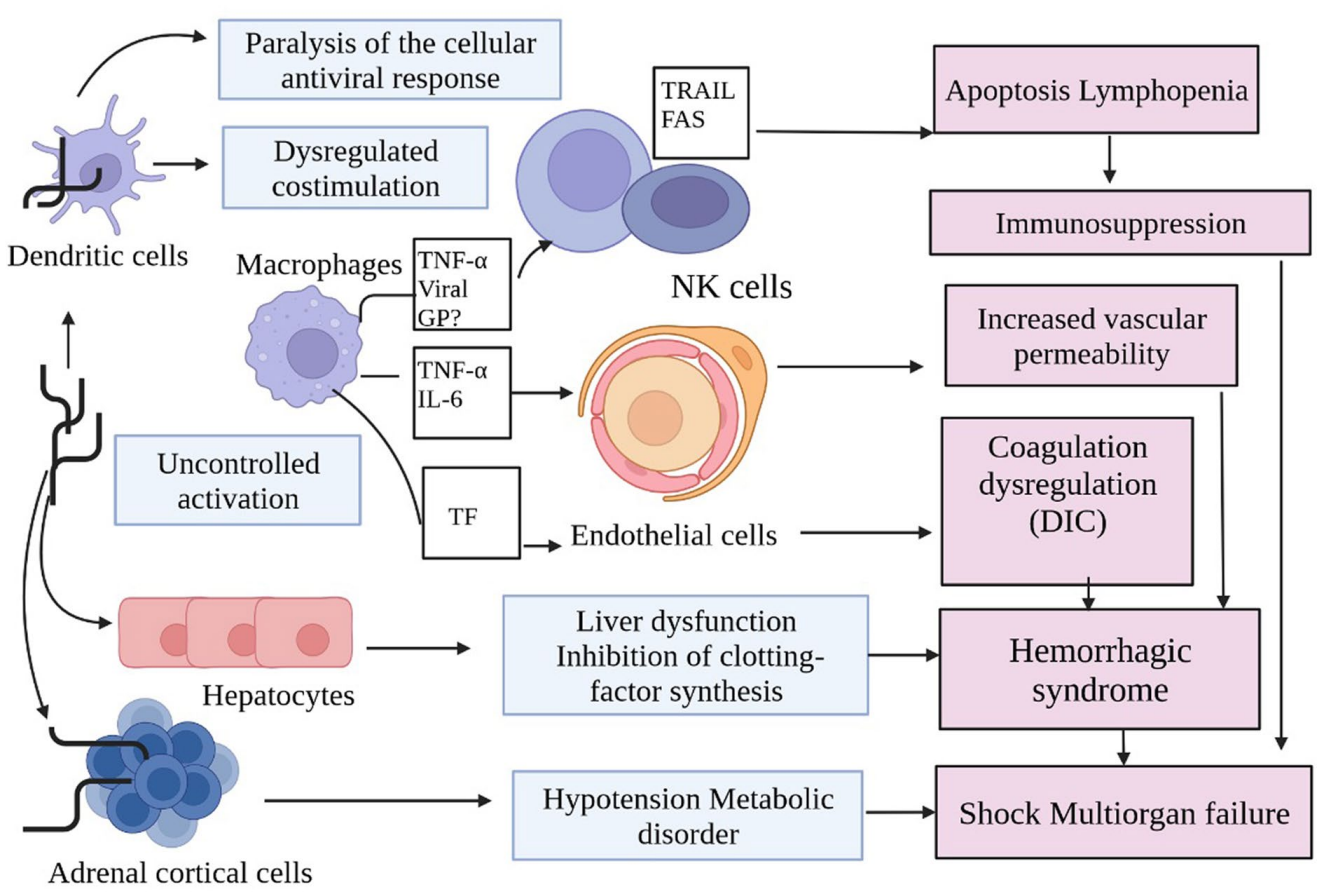
The pathogenesis of Marburg virus (MV) hemorrhagic fever in humans involves a complex sequence of interactions with various cell types. MV predominantly targets dendritic cells, monocytes, liver parenchymal cells, adrenocortical cells, and diverse lymphoid tissues. Dendritic cell infection results in compromised T lymphocyte stimulation, inducing lymphocyte apoptosis and subsequent immune suppression. This state amplifies cytokines/chemokines levels, culminating in shock and multiorgan damage. T lymphocytes and endothelial cells continue to suffer damage as a result of macrophage or monocyte infection, which sets off an unchecked cascade of cytokines and chemokines. Hemorrhaging is facilitated by endothelial cell infection, which increases blood vessel permeability and causes disseminated intravascular coagulopathy (DIC). Systemic replication may arise from endothelial cell infection. Parenchymal cell infection within the liver diminishes coagulation factors, potentially leading to later hemorrhage events. Infection of adrenocortical cells within the adrenal gland results in metabolic disturbances and dysregulated blood pressure, which can ultimately culminate in hemorrhage. Lymphoid tissue infections, especially those that affect the lymph nodes and spleen, cause tissue necrosis and impair adaptive immunity. In the later stages of the illness, shock and harm to the lymphoid organs can appear.

MVD models, and lab animals can all be used to study host pathophysiology and immune responses, as is widely recognized. Currently, NHPs (mainly cynomolgus and rhesus monkeys, African vervet monkeys, and baboons), hamsters, guinea pigs, and mice have been used to establish four MV disease models. NHPs are the “gold standard” among these models because to their great susceptibility to MV infections, which are almost always fatal, and their specific clinical traits that are like those of human infections. Furthermore, it has been demonstrated (Bente et al., 2009), that NHPs can directly transmit the MV virus through close contact. In the Kenyan case from 1987, MV infection was found in the peripheral blood mononuclear cell population of infected macaques using immunohistochemical, electron microscopy, and flow cytometric investigations (Mehedi et al., 2011). These studies also identified viral antigen and virions in both circulating and tissue-associated macrophages. Thus, the idea that the mononuclear phagocytic system—which consists of macrophages, monocytes, Kupffer cells, and dendritic cells—is the first cell type that MV infection targets—has been put forth (Brauburger et al., 2012). The lymph nodes, liver, and spleen all displayed the most severe necrotic lesions. Reticuloendothelial cells are abundant in the tissues, allowing infected cells to spread and infect more organs. At the organ level, the liver is a key site for MV replication, and the virus preferentially targets lymphoid tissues there (Shifflett and Marzi, 2019). There has been monocytoidal, plasma cellular alteration in the lymphatic tissue. Sites of necrosis also contain basophilic entities, either in the form of inclusion bodies in parenchymal cells or near necrotic cells. The other organs, on the other hand, are all affected by infection and show pathological changes, such as isolated or widespread necrosis without obvious inflammatory responses. Patients on MARD typically experience proteinuria, a symptom of renal failure. Grossly pale, swollen, and exhibiting significant parenchymal deterioration as well as indications of tubular insufficiency, the injured kidneys are also enlarged. Plasma cells and monocytes are prevalent in the mucous membranes of the stomach and intestines. Alveolar macrophages are found in hemorrhagic, obstructed alveoli in the lungs, and since fibrin surrounds them, these alveoli occasionally stain with viral antigen. In addition to necrosis (Shifflett and Marzi, 2019). The crimson pulp of the spleen, the lymph nodes’ follicles and medulla, as well as the lymphocyte count, are all noticeably necrosed in humans. Surprisingly, bystander apoptosis promotes lymphocyte loss rather than virus infection of cells. Aspartate aminotransferase, alanine aminotransferase, serum glutamic oxaloacetic transaminase, and serum glutamic pyruvic transaminase rise are liver tests that are suggestive of MV infection because the asialoglycoprotein receptor, a receptor specific to the liver, can boost these enzymes. Given that the liver produces multiple clotting factors, the pathological alterations to the liver are probably a contributing factor to the abnormalities in coagulation seen following MV infection. Severe MARD patients that undergo multiorgan failure do so as a result of the fatal virus’s increased mass effect. A decrease in the production of the steroid-synthesizing enzyme, involvement of the adrenal gland, and its failure all increase the risk of hypotension and hypovolemia, which finally result in shock (Figure 4; Mehedi et al., 2011). The symptoms of this toxic hemorrhagic fever, which have nothing to do with jaundice, include hemorrhagic diatheses in the skin and mucous membranes. Most of the histological abnormalities in the skin tissue include endothelial cell necrosis, localized hemorrhage, swelling, and variable degrees of cutaneous edoema. Using immunohistochemical stains, it is possible to identify various antigens in epidermal dendritic cells, endothelial cells, and connective tissue fibroblasts. The sebaceous and sweat gland epithelium also contains these antigens. Viral inclusions and particles can be observed inside endothelial cells and connective tissue using electron microscopy. Furthermore, it has been shown that the structural protein VP40 of the MV successfully aids in evading the host immune reaction to IFN (Martines et al., 2015).

### 6.1. Impact of MV on human organs

Multiple human organs may be harmed by the Marburg virus disease (MVD), which can result in organ failure and death (Abir et al., 2022). The liver and adrenal glands are the primary targets of the virus. Along with lymphocyte depletion, MVD can also harm lymphatic tissues by causing necrosis of the follicles, the medulla of lymph nodes, and the red pulp of the spleen. It is interesting to note that lymphocytes are not infected by the virus, despite the fact that bystander apoptosis causes a decrease in lymphocyte numbers (Mehedi et al., 2011; Abir et al., 2022). Monocytes and macrophages are two examples of mononuclear phagocytic cells that MV targets. By activating these cells, MV causes secondary targets like endothelial cells to get damaged. The advancement of shock, which is the main cause of death in MVD, is caused by the production of cytokines and pro-inflammatory mediators by activated macrophages and monocytes. The virus can also result in hemorrhagic signs, increased vascular permeability, and aberrant coagulation, all of which are interconnected and lead to the emergence of the classic symptoms of viral hemorrhagic fever (Mehedi et al., 2011; Abir et al., 2022). Orchitis (inflammation of the testicles) has been observed in male survivors, and central nervous system involvement can cause disorientation, irritability, and violence (Mehedi et al., 2011).

### 6.2. Diagnosis of MV

Due to the fact that the symptoms and signs of MVD overlap with those of endemic illnesses like Ebola and Lassa fever as well as infectious diseases like malaria, dengue fever, and typhoid fever, it can be difficult to diagnose. The fact that MVD symptoms cannot be present at first presentation and that fever may even go away entirely during the disease adds to its complexity (CDC, 2023a). This makes MVD prone to misdiagnosis, particularly outside of outbreak situations. Additionally, some women have presented with abortion as a manifestation of the disease, and pregnant females affected by MVD have shown higher mortality rates (CDC, 2023a; Roy et al., 2023). To properly manage the illness, a prompt and correct laboratory diagnosis is essential. PCR, immunoglobulin M Capture ELISA, and enzyme-linked immunosorbent assay (ELISA) are crucial confirming procedures for the diagnosis of MVD (Marburg Virus Disease). While IgG capture ELISA is used for late confirmation or finding recovered cases, the IgM capture ELISA is particularly helpful for early disease confirmation. Testing in Biosafety Level-IV laboratories is required for MVD diagnosis since handling the virus has a high level of risk (Islam et al., 2023b). To optimize efficiency and cost-effectiveness for the Ghanaian government, it is crucial to utilize advanced technologies and efficient transportation methods (CDC, 2023a; Roy et al., 2023). By implementing these measures, a limited number of laboratories can handle many samples quickly, while ensuring all necessary safety protocols are strictly adhered to. This approach will not only accelerate the diagnosis process but also enhance overall containment and protection against the spread of the virus.

## 7. Control and prevention of MV

To prevent the disease’s spread, the WHO has prescribed several measures to manage the virus (Mittler et al., 2018). When caring for patients with suspected or confirmed Marburg virus infections, healthcare professionals should take extra precautions to prevent infection. This means avoiding contaminated things and surfaces including soiled bedding and clothing in addition to the patient’s blood and bodily fluids. When near patients with MVD (within 1 m), healthcare staff should wear gloves, a clean, non-sterile long sleeve gown, and facial protection (a face shield or a medical mask and goggles). Residents of the affected areas should endeavor to educate the public about the signs and symptoms of the virus and the precautions that must be taken to prevent a pandemic. Healthcare professionals should think about building isolation units to immediately segregate MV-infected patients and stop person-to-person transmission (Brauburger et al., 2012). With the creation of accurate test diagnoses for suspected instances, the concept of halting transmission will also be strengthened even more. Contrary to previous epidemics, the deployment of barrier nursing techniques and training of hospital staff have benefited the general populace and decreased the incidence of nosocomial infections. Safe burial practices, sanitation standards, and public awareness campaigns are necessary to keep the virus under control since, as previously mentioned, close contact with the remains of infected people also contributes to the transmission of infection (Brauburger et al., 2012). Additionally, it is important to avoid direct contact with bodily fluids like blood, saliva, vomit, urine, and other bodily fluids from infected people. Additionally, care must be taken when handling vegetative items, such as infected needles and pins. It is also advisable to stay away from prospective carriers who are still alive and those who have passed away (such as monkeys, chimps, gorillas, fruit bats, and pigs; Government of Canada, 2023). Additionally, it is imperative that visitors to fruit bat colonies’ mines and caves wear gloves and other protective gear, including masks. The WHO further advises male MV disease survivors to practice safe sex and hygiene for 12 months from the onset of symptoms or until their semen has tested negative for MV twice (WHO, 2021a). Infection combat for Viral Hemorrhagic Fevers in the African Health Care Setting is a set of useful, hospital-based guidelines that the CDC and WHO have created in order to combat this deadly infection. Using locally accessible materials and minimal resources, this guideline intends to assist healthcare facilities in identifying cases and preventing the transmission of nosocomial diseases (CDC, 2023b). The European Network for Infectious Diseases (EUNID) outlines strategies for containing group 3 and 4 pathogen infections. Handling highly infectious diseases (HID) like MV requires cautious procedures in labs and hospitals. EUNID recommends initiating treatment for MV cases in high-level isolation units (HLIU), emphasizing isolation and cautious management. It advocates constructing specialized HLIUs for HID patients. Emergency departments must follow protocols for suspected MV cases. Laboratory personnel should inactivate samples, perform bedside tests, and adhere to safety measures. Intensive care for MV patients demands extra precautions and well-supported negative-pressure breathing apparatus. Careful management, including mask treatments and intubation, is crucial. Children with MV require careful handling to prevent spread. EUNID also offers guidelines for investigational interventions, emphasizing patient bedside procedures to minimize transmission risks (Brouqui et al., 2009).

### 7.1. MV’s surveillance strategy

The World Health Organization (WHO) advocates the implementation of vigilant surveillance for Marburg virus disease (MVD) as a preventive measure against outbreaks, alongside aiding countries at risk in formulating preparedness plans (WHO, 2021a). The subsequent recommendations, grounded in empirical evidence, delineate the optimal approaches for Marburg virus surveillance:

- Healthcare practitioners are advised to assess individuals exhibiting illness for the presence of Marburg virus disease, especially if they have been in close proximity to a person with suspected or confirmed MVD within the preceding 21 days or have visited an area with an ongoing Marburg outbreak during the same period (CDC, 2023c).
- Healthcare providers should exercise heightened vigilance and conduct evaluations for patients displaying symptoms suggestive of MVD (CDC, 2023d).
- Health care personnel tending to patients with presumed or confirmed Marburg virus infections are to implement supplementary infection control measures to avert contact with the patient’s blood, bodily fluids, and contaminated surfaces or items such as garments and bedding (WHO, 2023b).
- A variety of precautions can be taken by those who live in or travel to areas where the Marburg virus may be present to protect themselves and prevent the virus from spreading. These precautions include avoiding contact with the bodily fluids of those who are ill, delaying interaction with the semen of those who have recovered from MVD until the virus has been proven to be absent, and avoiding animals that may be carriers (CDC, 2023e).
- Marburg virus infection is categorized as a notifiable condition, necessitating the implementation of rigorous isolation precautions in instances of suspected infection. It is imperative to emphasize that Marburg virus disease is classified as a notifiable condition, mandating healthcare professionals to promptly report any confirmed case or suspected instances (CDC, 2023e).

### 7.2. Public awareness campaigns for MV

The Marburg virus infection can be addressed by departmental cooperation and public awareness efforts. Public education efforts can increase understanding of the disease’s severity and methods of prevention, influencing people to adopt healthy habits and seek the right medical care (Blasi et al., 2015). To effectively spread information and encourage safety precautions, these campaigns can make use of a variety of techniques, including media coverage, educational initiatives, and social campaigns (Raj, 2008; Akdim et al., 2021). In order to successfully use resources and knowledge, departmental coordination is crucial for a coordinated response to the disease (Kortepeter et al., 2020). The reach and impact of public awareness campaigns can be increased by cooperation across many ministries, including the Federal Ministry of Information, Federal Ministry of Health, and non-governmental organizations (Chagutah, 2009). Together, these initiatives can help to prevent, treat, and control the Marburg virus disease, thereby reducing its effects on people and communities.

### 7.3. Case studies in MV outbreak management

Several strategies are crucial in controlling Marburg virus (MV) outbreaks, as evidenced by various scientific studies and expert recommendations. Case isolation has been identified as a potent measure; a study published by the National Center for Biotechnology Information (NCBI) emphasizes that timely case isolation can effectively contain a MV outbreak (Ajelli and Merler, 2012). Successful outbreak control demands a multifaceted approach, involving case management, surveillance, and contact tracing, as advocated by the World Health Organization (WHO, 2021a). A robust laboratory service also plays a pivotal role in outbreak management, with WHO stressing its significance. Thorough evaluation of PCR-based methods, focusing on detection limits, proves essential for reliable diagnostics during monitoring phases (Timen et al., 2009). Ensuring safe and dignified burials for the deceased is another critical aspect of outbreak containment, as emphasized by WHO (2021a). Community engagement and social mobilization are integral to outbreak control. WHO underscores the importance of raising awareness about Marburg infection risk factors and protective measures to mitigate human transmission (WHO, 2021a). In terms of intervention, the development of effective vaccines, antivirals, and other therapeutic approaches, alongside appropriate mitigation strategies, emerges as paramount. This priority is affirmed by a bibliometric study published in Frontiers in Tropical Diseases (Islam et al., 2023a). Collectively, these strategies constitute a comprehensive framework for mitigating and controlling MV outbreaks.

## 8. Vaccine strategy of Marburg virus

The illness is caused by a virus that has no known cure and can only be passed from person to person or animal to animal through direct contact. Getting vaccinated is the greatest way to prevent the Marburg virus from spreading. Unfortunately, there is no Marburg virus vaccine available for use in the prevention of infection currently. Despite this, researchers have been working hard to create a vaccine for the Marburg virus, and several potential strategies have been assessed. Vaccination strategies, including the use of inactivated or destroyed Marburg virus particles, have been studied and assessed (Hunegnaw et al., 2021; Cross et al., 2022). This approach involves growing the virus in a petri dish and then killing it with heat or chemicals. The inactivated viral particles are then employed to create a vaccine. Vaccines against numerous viruses, including polio and hepatitis A, have been successfully developed using this method. One approach that has been studied is the use of a live-attenuated strain of the Marburg virus as a vaccine. This method involves making the virus less infectious or virulent by laboratory manipulation. After then, the engineered virus is used to make a vaccine. Measles and mumps can be prevented by the use of vaccines that include reduced forms of the infectious agent. The third sort of vaccination strategy that has been researched is the use of vaccines that are based on viral vectors. The core of this method is to deliver genetic material from the Marburg virus into the body via a non-pathogenic virus. An immune response will be triggered by the presence of the genetic material, protecting the body from the Marburg virus (Matassov et al., 2018; Cross et al., 2020). Infectious diseases like Ebola can be prevented and treated by vaccines that use viruses as “vectors.” A fourth method that has been tried and tested is the use of protein-based vaccines. The purpose of this method, which involves the use of a protein isolated from the Marburg virus, is to provoke an immune response. Vaccines frequently include the protein, which has been created using recombinant DNA technology. Protein-based vaccines have proven to be effective, leading to the development of preventative measures against diseases like human papillomavirus (HPV). Vaccines against Marburg virus are currently in development, although there are other choices. Possible vaccine targets include the use of vesicular stomatitis virus (VSV) as a vector, the VSV-EBOV-MV vaccine is a viral vector-based vaccination that protects against both Ebola and Marburg (Woolsey et al., 2022). The vaccine is currently being tested in clinical settings after displaying encouraging outcomes in preclinical research. The Ad26.ZEBOV/MV vaccine delivers antigens against the Ebola and Marburg viruses using an adenovirus as a vector. The NIH oversees developing this vaccine. After showing promising results in preclinical tests, the vaccine is currently being studied in clinical settings. The vesicular stomatitis virus (VSV) serves as a vector to transmit genetic material from the Ebola and Marburg viruses for the rVSVG-ZEBOV-GP/MV-GP viral vector-based immunization. Researchers from the University of Maryland created this vaccination. The vaccine is currently being tested in clinical settings after displaying encouraging outcomes in preclinical studies. A Marburg virus vaccine known as mRNA-1,360 creates an antiviral protein using mRNA technology. The vaccine is currently being tested in clinical settings after displaying encouraging outcomes in preclinical studies. The Marburg Vax vaccine is a dead virus-based immunization since it is made from inactivated Marburg virus particles. Its main goal is to provide protection (Jones et al., 2005; Marzi et al., 2019, 2021). The vaccine is currently being tested in clinical settings after displaying encouraging outcomes in preclinical studies. The creation of a Marburg virus vaccine is essential for stopping future epidemics. Although there are many ways to make vaccines, the relevant authorities have not yet approved any treatments or vaccines for MV. Prior to the 2013–2016 EBOV pandemic, there was a paucity of significant public sector investment for the development of pharmaceutical countermeasures, despite the catastrophic effects of filovirus infections on public health. However, funding for basic and translational studies of filoviruses has increased since 2016 as a result of biodefense and research grants. As a result, the licensing procedure for EBOV vaccines and medications to be used in suppressing outbreaks has advanced. With the help of this financing, MV was able to expand its study of animal models and preventative measures, both of which are essential before the start of clinical testing. In NHP research, several approaches have showed promise, and these approaches are now being developed via Phase I clinical trials for the clinical development of vaccines and antivirals (Marzi et al., 2015; Krause et al., 2020). Since almost the moment the virus was identified, a MV vaccine has been under development, but results have been patchy. Only a few of the prospective MV vaccination platforms have shown protective efficacy in NHPs, despite being extensively tested in rodent models. The MV glycoprotein (GP) is used by all presently available successful vaccine candidates as their primary antigen. It offers defense against a variety of RAVV and MV strains. As MV vaccines, fast-acting, live-attenuated, non-replicating, and replicating viral vector regimens may be utilized in multidose, single-dose, and other forms. In the event of an outbreak, reactive vaccination campaigns would make use of single-dose, quick-acting vaccinations. There are, however, a number of candidate vaccines that could be employed for at-risk groups’ regular vaccination. The duration of the acquired immunity created by the vaccine will determine whether additional immunizations are required (Dean et al., 2020; WHO, 2023c).

### 8.1. Vaccine status and WHO guideline

The two-day-old vaccine that prevents illness is supported by the National Institute of Health. In this investigation, a deadly dose of the Marburg virus vaccine was given to rhesus macaques. After 48 h, just two of the five to six monkeys that had received the vaccine were still alive (Geisbert, 2015). These infections can only be treated in laboratories with the utmost level of safety and containment because there are not any licensed immunizations that have been approved for use in humans. As indicated in Table 3 (Grant-Klein et al., 2015), several vaccination approaches had been developed for the treatment of MV in NHP models. There is not a licensed vaccination or antiviral medication for MVD now. Supportive therapy that restores lost blood and clotting elements while also preserving blood pressure, oxygen levels, and electrolyte balance may be helpful. There are several MV vaccines developed. For instance, the VSV-MV vaccine, a recombinant VSV-based immunization that produces the MV glycoprotein, instantly shields hosts from MVD in animal models (Marzi et al., 2021). Another strategy for prevention against both hemorrhagic viruses is the vaccine candidate MVABN-Filo, which contains antigens from the Marburg and the Ebola viruses (Barry et al., 2021). A phase 3 trial that appears to create a powerful defense against the Ebola virus is currently being carried out. In addition to developing preventive vaccines, researchers are currently striving to develop effective postexposure therapies for MVD, such as small molecule antivirals and monoclonal antibodies (mAbs) specific to the MV. Researchers combined a monoclonal antibody (MR186-YTE) with an antiviral (remdesivir) using a non-human primate model of MVD (Cross et al., 2021). The statistics show that this combination was very effective in treating the condition. A summary of the advancement of MVD vaccines and experimental therapies is shown in Table 4 (Baby et al., 2022).

**Table 3:** Vaccine efficacy on the non-human primate model.

S. no.	Vaccine type	Dose	Survival %	References
1	Virus like particles + RIBI (Vaccine adjuvant)	3	100	Hevey et al. (1998)
2	EBOV GP + SUDV GP + MV GP + RAVV GP	3	100	Ashique et al. (2023)
3	rAD5 (vector) + MV GP + DNA MV GP	4	100	Barry et al. (2021)
4	Intact MV, RAVV	2	50	Swenson et al. (2004)
5	VEEV + MV GP + VEEV-MV NP	3	67–100	Marzi et al. (2021)
6	Cad Vax-Pan Filo	2	100	Cross et al. (2021)
7	MV GP	3	67	Geisbert (2015)
8	VSV + MV	1	100	Grant-Klein et al. (2015)

**Table 4:** Development of a vaccine and advancements in experimental therapies for Marburg virus sickness.

Vaccine	Description	Animal studies	Clinical trials	References
MVA-BN-Filo	A multivalent vaccination formulation called Mvabea (MVA-BN-Filo) is intended to give active acquired immunity to the Sudan virus, Ebola virus, Marburg virus, and Tai Forest virus.	No data.	Eight months after immunization, phase 1 results showed persistent GP immunity against Ebola.	Marzi et al. (2015)
			No MV-related findings were reported.	
rVSV-MVGP	A vesicular stomatitis virus vector that is recombinant and expresses the MV glycoprotein (GP).	100% of animal studies are successful. One year after the shot	No data.	Henao-Restrepo et al. (2017)
cAd3-MV	In this immunization, a modified chimpanzee adenovirus known as cAD3 is utilized.	No data for cAd3-MV	Four weeks following a single vaccination, 95% of individuals exhibited a glycoprotein-specific antibody response, which persisted in 70% of them at 48 weeks.	Dean et al. (2020)
CAdVax-panFilo	The complex encodes GPs from EBOV, SUDV, and MV as well as GPs from EBOV and MV Musoke nucleoproteins.	Antibodies were tested against all five filoviruses, and no macaques displayed symptoms of a clinical disease.	No data.	Dean and Longini (2022)
DNA plasmid vaccine	MV Angola DNA is expressed by a Marburg DNA plasmid.	A DNA prime/boost vaccine in macaques provided protection, although all of the animals eventually became ill.	In a phase 1 experiment, 10 participants showed 90% antibody responses.	Cross et al. (2022)

## 9. T-705 and remdesivir: promising antiviral strategies for Marburg virus disease

Two antiviral agents, T-705 (favipiravir) and remdesivir, have exhibited efficacy against Marburg virus disease (MVD) through preclinical investigations. These agents function as RNA polymerase inhibitors, impeding viral replication and transcription processes (Furuta et al., 2013; Bixler et al., 2018; Marlin et al., 2022). T-705 (favipiravir), a pyrazinecarboxamide derivative, boasts comprehensive antiviral activity across diverse viruses and holds clinical approval in Japan for addressing influenza (Zhu et al., 2018; Zadeh et al., 2022). A research study disseminated by the National Center for Biotechnology Information (NCBI) proposes T-705 as a viable therapeutic candidate against Marburg virus, particularly valuable for outbreak scenarios due to its prompt and secure oral administration following exposure (Zhu et al., 2018; Zadeh et al., 2022). Angola, a mouse-adapted Marburg virus strain, was used to infect mice in a different investigation, and T-705 showed excellent survivability in these mice (Zhu et al., 2018; Zadeh et al., 2022). Oral dosing that began 1 or 2 days after infection and continued for 8 days resulted in full mouse survival. Remdesivir functions as a monophosphoramidate nucleoside prodrug, which undergoes intracellular metabolic alteration into an active nucleoside triphosphate form, thereby inhibiting viral RNA polymerase (Malin et al., 2020). Notably, a study revealed the curative effectiveness of remdesivir in nonhuman monkeys infected with the Marburg virus experimentally, particularly when therapy started 5 days after inoculation (Porter et al., 2020). Remdesivir was demonstrated to be effective against a variety of RNA viruses *in vitro* and using macaque models, including the Ebola virus, Lassa virus, and Marburg virus (Malin et al., 2020). This research was published in Clinical Microbiology Reviews. It is imperative to acknowledge that the efficacy and safety of these agents in human MVD cases remain untested. Moreover, the application of antiviral drugs necessitates integration with supplementary control measures, including case isolation, surveillance, contact tracing, laboratory services, secure burials, and community mobilization, to ensure the effective management of MVD outbreaks (Cross et al., 2018; Zhu et al., 2018). T-705, also known as Favipiravir, has demonstrated effectiveness *in vitro* and *in vivo* against Marburg virus infection (Zhu et al., 2018). Mice that had been intraperitoneally infected with Marburg virus that had been modified for mice completely survived when given T-705 beginning 1 or 2 days after infection and continuing for 8 days (Zhu et al., 2018). Vero E6 cells showed no negative effects (Zhu et al., 2018). A broad-spectrum antiviral drug called Remdesivir has previously shown antiviral effectiveness against filoviruses including the Marburg virus (Levien and Baker, 2021). Respiratory failure and organ malfunction, including low albumin, low potassium, low red blood cell count, low platelet count, which aids clots, and yellow skin coloring, are the most frequent side events in Remdesivir trials for COVID-19 (Zadeh et al., 2021). Pyrexia, sleeplessness, multi-organ malfunction, DVT, and hypersensitivity/anaphylactic reactions connected to the infusion are additional negative consequences (Levien and Baker, 2021). A combination therapy of monoclonal antibodies (mAbs) and remdesivir has been shown to induce an 80% protection rate against Marburg virus in rhesus macaques (Cross et al., 2021; Rees, n.d.). The combination therapy was initiated 5 days post-inoculation with Marburg virus (Cross et al., 2021). High temperature, severe headaches, severe malaise, muscle aches and pains, gastrointestinal problems, migraines, and dizziness are all signs of Marburg virus sickness (Kortepeter et al., 2020; WHO, 2021b,c). Marburg virus sickness has a case fatality ratio that can reach 88%, but it can be significantly reduced with proper patient care (WHO, 2021b). In summary, Remdesivir has shown antiviral activity against filoviruses like Marburg virus, whereas T-705 has showed efficacy against Marburg virus infection *in vitro* and *in vivo*. In rhesus macaques, monoclonal antibody and remdesivir combination therapy has been reported to result in an 80% protection rate against Marburg virus. High temperature, severe headaches, severe malaise, muscle aches and pains, gastrointestinal problems, migraines, and disorientation are all signs of Marburg virus sickness.

## 10. Emerging therapeutic agents in clinical trials

Clinical trials have been conducted for some drugs to treat MV; some of these trials have been completed and others are ongoing. MV drug development has progressed much more slowly than preclinical and clinical trials for the treatment of EBOV. Antiviral effectiveness against MV infections in nonhuman primates (NHPs) has long been regarded as the gold standard to assess prospective efficacy in humans and support subsequent clinical trials, much like EBOV. Following exposure in NHPs with advanced disease, several promising strategies, such as pan-filoviral small-molecule antivirals and MV-specific monoclonal antibodies, have shown excellent success. Table 5 shows the biologics currently being tested in clinical trials, along with their current status and research center location (Citeline Clinical, 2023).

**Table 5:** Ongoing clinical trials for therapeutics.

S. no	Research study	Research place	Disease	Interceding	Status of trial	References
1.	Healthy Adults Receiving the CAd3-Marburg Vaccine	WRAIR-clinical trials center, silver spring, US	Marburg virus	Biological: cAd3 vaccine	Completed	Ashique et al. (2023)
2.	Single Administration Safety Study	American West Coast, California	Marburg Hemorrhagic Fever	Drug:AVI-6003	Completed	Ashique et al. (2023)
	Treatment for the Marburg Virus Post-exposure Prophylaxis			Drug: Placebo		
3.	Filo and Ad26 from MVA-BN(R). On healthy volunteers, ZEBOV vaccines are being tested for safety.	Atlanta, Georgia, US	Marburg Hemorrhagic Fever	Biological: Ad26 Zaire Ebola Vaccine.	Completed	Ashique et al. (2023)
			Ebola virus disease	Biological: MVA Multi-Filo Ebola Vaccine		
4.	An investigation on the safety, tolerability, and pharmacokinetics of a single dose of IV BCX4430	US-Kansas City, Lenexa, PRA Health Sciences	Marburg Virus	Drug: Galidesivir	Active, not recruiting	Ashique et al. (2023)
				Drug: Placebo		
5.	Avi-7,288 Pharmacokinetics, Safety, and Tolerability Study in Healthy Adult Volunteers	Clinical Pharmacology Center at SNBL. USA, Baltimore	Marburg Hemorrhagic Fever	Drug: AVI-7288	Completed	Ashique et al. (2023)
				Other: Placebo		
6.	Marburg and Ebola virus vaccines	9,000 Rockville Pike, National Institutes of Health Clinical Center, Bethesda, Maryland (USA)	Marburg Virus Illness	Drug: VRC-EBODNA023-00-VP	Completed	Ashique et al. (2023)
			Ebola Virus Illness	Drug: VRC-MARDNA025-VP		

## 11. Conclusion

The emergence of Marburg virus (MV) has presented a complex global challenge characterized by fatal outbreaks and significant clinical hurdles. The extensive analysis conducted in this study sheds light on the multifaceted nature of MV, encompassing its origin, transmission dynamics, clinical manifestations, and the intricate interplay between the virus and its human hosts. The documented outbreaks underscore the urgency of understanding and effectively addressing the threat posed by MV to global health security. Throughout this investigation, it has become evident that the clinical challenges posed by MV are substantial, with its virulent nature leading to high mortality rates and a range of severe symptoms. The lack of specific antiviral therapies or vaccines further amplifies the urgency of comprehensive prevention and control strategies. The cases studied emphasize the need for rapid, coordinated responses involving healthcare providers, researchers, and governmental bodies to curtail the impact of MV outbreaks. The global perspective on MV’s emergence emphasizes the need for proactive, multidisciplinary approaches to prevent and mitigate its devastating impact. The lessons learned from past outbreaks underscore the importance of preparedness, collaboration, and innovation in addressing this ongoing threat to public health. Through these concerted efforts, we can hope to avert future MV outbreaks and minimize their toll on human lives.

## 12. Future perspectives

Looking ahead, several crucial research and intervention directions emerge from our analysis. First, continued efforts are imperative to elucidate the intricate mechanisms of MV transmission from its reservoir hosts to humans, allowing for the identification of potential intervention points. Collaborative studies integrating virology, epidemiology, and ecology will be pivotal in achieving a comprehensive understanding of the virus’s life cycle. Second, the development of effective preventative measures remains paramount. Advances in vaccine technologies offer promise for the creation of MV-specific vaccines, analogous to strategies employed against related pathogens. In parallel, research should focus on identifying small molecule antiviral compounds that can impede MV replication and spread. Third, bolstering healthcare infrastructure in regions susceptible to MV outbreaks is essential. Strengthening diagnostic capabilities, training healthcare workers in effective infection control measures, and establishing rapid response protocols are vital components of managing MV cases and preventing wider disseminations.

## Author contributions

SSr, DS, SK, and AS: concept and original draft. RR, AA, SA, SR, SSa, ZA-q, PB, AM, AR-M, and RS: reviewing and editing. RS: supervision. All authors contributed to the article and approved the submitted version.

## Conflict of interest

The authors declare that the research was conducted in the absence of any commercial or financial relationships that could be construed as a potential conflict of interest.

## Publisher’s note

All claims expressed in this article are solely those of the authors and do not necessarily represent those of their affiliated organizations, or those of the publisher, the editors and the reviewers. Any product that may be evaluated in this article, or claim that may be made by its manufacturer, is not guaranteed or endorsed by the publisher.

## Abbreviations

CIRMF: Centre Interdisciplinaire de Recherches Médicales de Franceville
DRC: Democratic Republic of Congo
EBOV: Ebola virus
EUNID: European Network for Infectious Diseases
GP: Glycosylated protein
HLIU: High-level isolation unit
HPV: Human papillomavirus
IFN: Interferons
L: Large protein
MV: Marburg virus
MVD: Marburg virus disease
NHPs: Non-human primates
NIAID: The National Institute of Allergy and Infectious Diseases
PPE: Personal protective equipment
RT-PCR: Real-time polymerase chain reaction
VSV: Vesicular stomatitis virus
WHO: World Health Organization
